# Supplementary material for: Hepatocyte Growth Factor Enhances Antineoplastic Effect of 5-Fluorouracil by Increasing UPP1 Expression in HepG2 Cells
Source: Int J Mol Sci. 2022 Aug 14;23(16):9108. doi: 10.3390/ijms23169108 (PMC9409026; doi:10.3390/ijms23169108)
Supplement: Supplementary file 1 [file ijms-23-09108-s001.zip › Supplementary Figure S1.pdf]

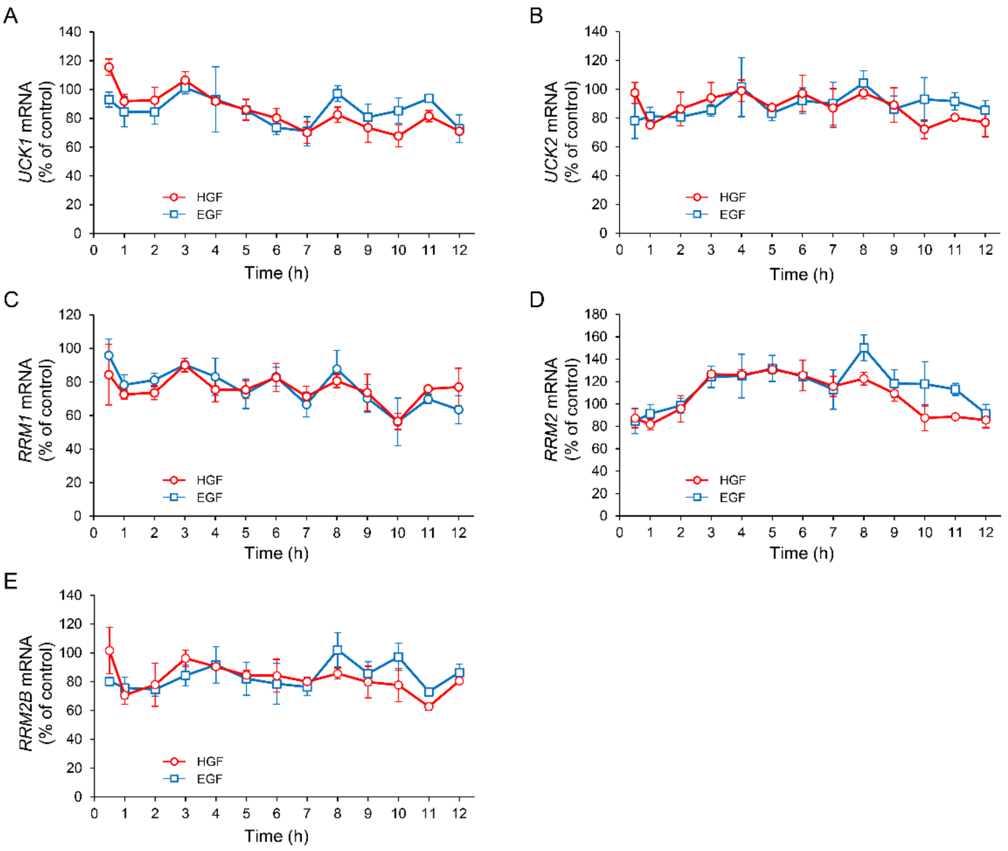

Figure S1. Effects of HGF and EGF on mRNA expression of *UCK1*, *UCK2*, *RRM1*, *RRM2*, and *RRM2B*. HepG2 cells were cultured for 24 h in serum-starved medium, and incubation was continued after addition of HGF (50 ng/mL) or EGF (50 ng/mL) for the indicated period. Thereafter, total RNA was extracted. The transcription levels of *UCK1* (A), *UCK2* (B), *RRM1* (C), *RRM2* (D), and *RRM2B* (E) are expressed as a percentage of that in cells not treated with growth factors at 0 h. Each bar represents the mean ± SD of 5 experiments.
