# Supplementary material for: Hepatocyte Growth Factor Enhances Antineoplastic Effect of 5-Fluorouracil by Increasing UPP1 Expression in HepG2 Cells
Source: Int J Mol Sci. 2022 Aug 14;23(16):9108. doi: 10.3390/ijms23169108 (PMC9409026; doi:10.3390/ijms23169108)
Supplement: Supplementary file 1 [file ijms-23-09108-s001.zip › Supplementary Figure S2.pdf]

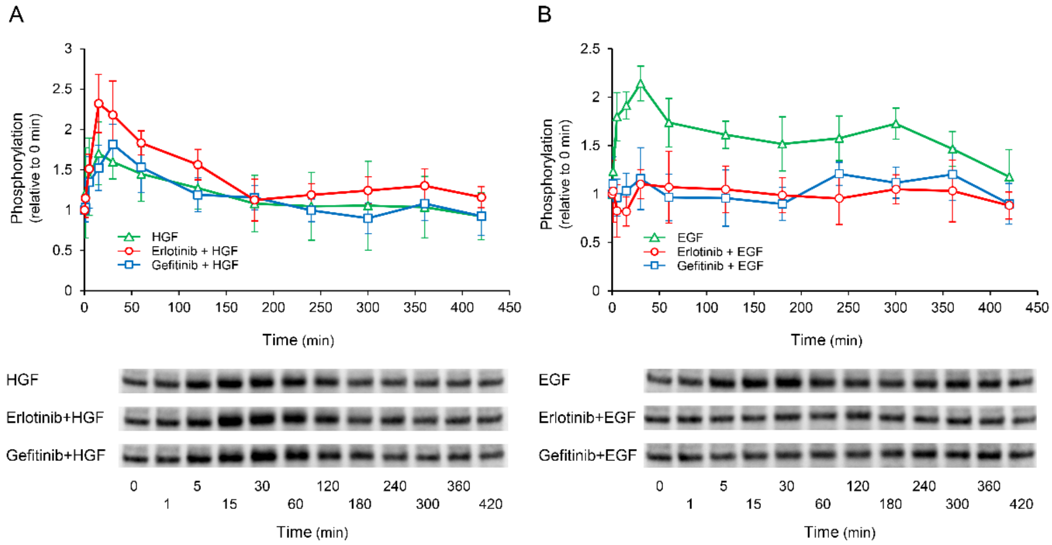

Figure S2. Effects of erlotinib and gefitinib on growth factor-induced phosphorylation of Akt1/2. HepG2 cells were cultured for 24 h in serum-starved medium. Thereafter, HGF (50 ng/mL: (A)) or EGF (50 ng/mL: (B)) was added to the medium. Cells were pretreated with erlotinib (5 μM) or gefitinib (1 μM) 15 min before treatment with growth factors. Total protein was collected at the indicated time points. The relative amount of phosphorylated protein in each band was quantified by ImageQuant TL software, and phosphorylation trends of Akt1/2 after treatment with growth factors were plotted on graphs. The intensity of phosphorylation is expressed relative to that at 0 min. Western blot analysis was performed in 3 experiments for 3 different preparations, and representative blots are shown. Each bar represents the mean ± SD.
